# Apology and Bills

**Published:** 1858-08

**Authors:** N. S. Davis

**Affiliations:** Proprietor


RUSH MEDICAL COLLEGE.—Clinical instruction and dissections will commence in connection with this institution on the first Tuesday in October. The Hospital is well filled with patients, and quite a class already in attendance three times a-week. The material for dissection will be abundant. A course of preliminary lectures will also be given during the month of October. The prospects for a good class, and the preparations for their instruction were never better.

